# Developing BIOTEL: A Semi-Automated Spreadsheet for Estimating Telomere Length and Biological Age

**DOI:** 10.3389/fgene.2019.00084

**Published:** 2019-02-19

**Authors:** Aristidis Tsatsakis, Dimitrios Tsoukalas, Persefoni Fragkiadaki, Elena Vakonaki, Manolis Tzatzarakis, Evangelia Sarandi, Dragana Nikitovic, Gerasimos Tsilimidos, Athanasios K. Alegakis

**Affiliations:** ^1^Laboratory of Toxicology, Medical School, University of Crete, Heraklion, Greece; ^2^Metabolomic Medicine, Health Clinics for Autoimmune and Chronic Diseases, Athens, Greece; ^3^Laboratory of Anatomy-Histology-Embryology, Medical School, University of Crete, Heraklion, Greece

**Keywords:** telomere length, spreadsheet, biological age, BIOTEL, aging

## Abstract

**Introduction:** Telomere length (TL) is causally related to aging and several age-related diseases. Specifically, the abundance of short telomeres and the rate of telomere shortening are strong determinants of cell homeostasis. Thus, tools for analyzing and manipulating TL data can vastly improve research focused on aging. Aim: In this study, we developed a semi-automated worksheet, BIOTEL, to generate individual and group TL statistics and provide a crude estimation of biological age.

**Results:** Data from the Telomere Length Database Project (TLDP) were implemented to the spreadsheet to produce TL statistics. 150 participants were included, and their age was from 21 to 82 years, and the sex distribution ratio was 52.3%: 47.7% (male: female). Initially, we analyzed the fluorescence intensities of telomeres that were measured on metaphase spread leukocytes using three-dimensional (3D) quantitative-fluorescent *in situ* hybridization (Q-FISH) procedures (3D DNA FISH) with a (C3TA2)3 peptide nucleic acid (PNA) probe. Raw data of fluorescence intensities, demographic data and medical records from the participants were imported into the worksheet. Basic statistical analyses of TL data were provided through BIOTEL, including TL percentiles, specialized charts for TL distribution including the percentage of critically short telomeres (< 3,000 kilobases), individual telomere profiles, and graphs of biological age vs. chronological age.

**Conclusion:** BIOTEL ver. 2.4 is a functional semi-automated worksheet that calculates a wide range of TL statistics, thus a useful tool with applications in research of telomeres and biological age estimation.

## Introduction

Telomeres are terminal DNA-protein complexes that protect chromosomes and, in collaboration with sheltering proteins, help maintain genomic stability (Palm and de Lange, 2008). In adult somatic cells, telomeres shorten every time cells divide, resulting in progressive shortening with age. Thus, telomere length (TL) is a useful marker of biological aging (Blackburn et al., 2006). Monitoring biological age can be used to assess the aging process and to determine how external factors influence aging rates. Indeed, several studies have examined associations between TL and chronological age-associated traits. Thus, in a review of 124 cross-sectional studies in adults, Müezzinler et al. (2013) reported a negative correlation of about 0.3 between age and both absolute and relative TL. The largest population-based TL study to date (*n* = 105,539) concluded that women, on average, have longer telomeres than men, suggesting that women exhibit lower biological age (Lapham et al., 2015). However, differences in biological age for individuals of the same chronological age independent of sex have been reported.

Furthermore, TL is correlated with obesity, cancer, diabetes, and cardiovascular disease (Marioni et al., 2016). To date, few studies have examined longitudinal telomere change, and those few studies were carried out in small population sets and a few time points (Müezzinler et al., 2013). Thus, further studies with large datasets and TL statistics are needed to determine the association between TL and biological age.

Microsoft EXCEL^®^ is a widely used and well-known program. EXCEL is particularly useful for basic and non-demanding precision numeric calculations, with several advantages in statistical analysis including (a) special validation and formatting processes like Data Validation and Conditional Formatting, (b) easy in handling repeating data (e.g., drag and drop features), (c) exporting and importing data to other statistical programs (e.g., SPSS Statistics), d) the inclusion of basic statistical and other type functions, and (e) ease in producing graphs. However, EXCEL also has certain disadvantages including (a) accuracy estimations (McCullough and Wilson, 2005; Almiron et al., 2010), (b) programming [Visual Basic for Applications (VBA) routines], and (c) limitations in capacity (16,384 columns) and the number of records (1,048,576 rows).

Several spreadsheets have been developed in behavioral science and the research field of medicine and biology (Dubuque, 2015; Theodorsson, 2015; Carden et al., 2017; Deochand, 2017). Indeed, spreadsheets used in genomics and bioinformatics research have been generated through EXCEL (Fredriksson et al., 2014; Niedz, 2016). However, there have been several reports of false gene identification associated with the utilization of specific spreadsheets (Zeeberg et al., 2004).

Researchers that analyze telomeres use mostly software that employs image processing and data processing of telomere measurements. One such example is the application of an open-source Java program, Telometric, which has been reported to provide an overestimation of mean TL when processing agarose gel images (Grant et al., 2001; Haussmann et al., 2011). MeTelenisa freeware software with special options like correction of irregular light effects and elimination of background fluorescence in Q-FISH images and has been used widely (Barkovskaya et al., 2016). On the other hand, Computel estimates mean TL from the whole genome based on sequencing data (Nersisyan and Arakelyan, 2015). In the same category, TFL-TELO can be applied to FISH images and provide information of telomeres from single chromosomes. However, the manipulation of fluorescence intensities data is challenging, and the accuracy of these measurements was assessed by Poon et al. (1999). Finally, TeloTool is a Matlab toolbox applied in TRF analysis with sophisticated approaches in estimation but cannot perform a wide variety of statistical analysis (Göhring et al., 2014). To our knowledge, there is no specialized software to assess the telomere profile of an individual or group and provide accurate statistics (means, percentile) and graphs.

In the present study, we developed a semi-automated spreadsheet (BIOTEL) as a user-friendly application to assess TL raw data and generate TL statistics, which can be used to estimate biological age and produce specialized charts and reports. Our results demonstrate that BIOTEL can be used to calculate TL for an individual or group efficiently, assess the telomere change and provide TL measurements for a TL vs. age nomogram.

### Implementation

BIOTEL, from the Greek word “bio” and the initials from the word “telomere,” was developed utilizing a spreadsheet program (EXCEL 2007). BIOTEL uses standard functions and procedures installed in EXCEL, including the Pivot Tables and Name Manager procedures, but also utilizes mathematical, lookup, date, and text functions such as AVERAGE(), PERCENTILE(), YEAR(), and LEFT(). There are no user-written VBA procedures, and users must install the Analysis Tool Pack and Lookup Wizard from the add-ins EXCEL pack. For this study, BIOTEL was implemented with data from the Telomere Length Database Project (TLDP), a developing TL research database.

#### Data Collection

**FIGURE 1:**
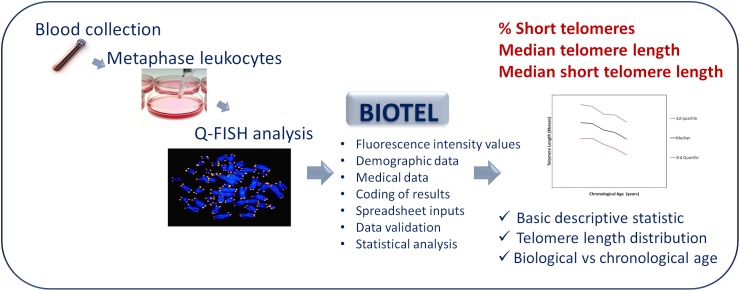
An overview diagram of the telomere estimation process. Q-Fish analysis on metaphase leukocytes, isolated from human blood was applied. BIOTEL combine the fluorescence data of leukocyte telomeres with demographic and medical data. After coding and validation of data, estimation of statistical measure and graphical representations of data were provided.

The diagram showing the process of telomere length estimation is depicted in Figure 1. Briefly, participants were selected from patients admitted to a private clinic in Athens and from personnel of the University Hospital of Heraklion. Demographic data and medical history of chronic diseases were obtained. Demographic data were name, surname, date of birth, date of sample collection, and sex. Medical history included all the self-referred statements for chronic diseases such as diabetes, chronic obstructive pulmonary disease, cancer, and rheumatoid arthritis.

Moreover, possible exposure to pesticides (occupational) and use of antidepressants was also noted. According to the above criteria, 150 participants were included in the TLDP database. The age of the participants spanned from 21 to 82 years, and 52.3%of them were male. No participants suffered from any chronic diseases or took any medication or supplements.

#### Q-Fish Analysis

Peripheral blood samples of 2.5 ml were collected from all participants. Heparinized blood was cultured in RPMI1640 culture media supplemented with 10% fetal bovine serum, 1% L-Glutamine, 1% penicillin and streptomycin and stimulated for 72 h in a CO_2_ incubator with phytohemagglutinin. For metaphase chromosome preparations, 10 μg/ml colcemid was added to each culture 2 h before harvesting, followed by KCl hypotonic shock and methanol/acetic (3:1) fixation.

A few drops of fixative solution containing cells were used for each slide. Slides were dried and incubated on a hot plate (55°C) overnight before hybridization. Slides with chromosome preparations were hybridized with a (C3TA2)_3_ peptide nucleic acid (PNA) probe labeled with Cy3 (PANAGENE). Twenty micro liters of hybridization mixture containing 60% formamide, 0.3 μg/ml Cy3 (C3TA2)3 PNA probe in 20 mM Tris pH 7.4 was added to the slide, a cover slip (76 × 26 mm) was added and DNA was denatured by heat for 10 min at 85°C. After hybridization for 2 h at room temperature, the slides were washed at 550°C with washing solution containing 0.05% Tween-20 (2 × 10 min). The slides were then stained with DAPI solution (0.5 μl in 1 ml SSC) for 20 min and were washed with SSC solution (2 × 2 min). The slides air dried and covered by mounting media and a cover slip before proceeded with images acquisition.

After hybridization, images from metaphase FISH experiments were acquired using inverted laser scanning confocal microscope with spectral detection (Leica TCS SP8 microscope unit). In total 20–30 optical section were captured with a step of 250 nm. Images were captured for each slide, using a 63 × objectiveand a charge-coupled device camera at 1024 × 1024 pixels resolution and 8 bit depth. A 405 nm laser was used to excite and detect DAPI for nucleus staining, while a 568 nm laser was used to excite and detect the telomere (Alexa Fluor 647). In order to avoid differences between the replicates, the settings for exposure and gain remained constant between captures. For each slide more than 10 different scanned images were obtained from 3 independent biological replicates. Maximum projections and deconvolution of the images were performedwith the Leica Q-FISH software, and telomere fluorescence intensity was analyzed using Image J^1^ by two independent investigators.

Two levels of calibration were used to ensure a reliable quantitative estimation of telomere length in the various samples. First, to correct for daily variations in lamp intensity and alignment, images of fluorescent beads (orange beads, size 0.2 μm, Thermo Fisher Scientific) were acquired just prior to acquisition of the images from the samples. The fluorescence intensities of the beads and telomeres were analyzed using Image J. Second, telomere fluorescence intensity values were normalized between slides, using the mean for intensity values of L5178Y-S that had been included as calibration standard in each experiment. Telomere fluorescence values were converted into kb by using L5178Y-S cells with stable and known telomere length which was estimated to be around 7 kb (McIlrath et al., 2001).

#### Description of BIOTEL

BIOTEL is an EXCEL worksheet used to estimate TL. It is a standalone program, although the central insight is to develop additional software for all the steps of telomeric analysis, from image analysis to data reports. Implementation of BIOTEL was started in Microsoft Office EXCEL 2010 (Windows operating system), but it was also tested in an earlier version 2007 and a post-version EXCEL 365. BIOTEL consists of 10 sheets. The first sheet, “MAIN,” is for navigation between sheets using hyperlinks. The second sheet, “PARAMS,” comprises information from the Q-FISH telomere analysis such as the cell series fluorescence and date of cell line analysis. Tips on BIOTEL handling, data names, and data ranges were also included in this sheet.

The third sheet, “DEMOGRAPHICS,” includes participant demographic data, dates of birth, sampling and Q-FISH analysis, the existence of chronic diseases, occupational exposure to pesticides, use of illicit drugs, and data on repeated measurements (1st, 2nd, 3rd repeat).

The fourth sheet, “ILLUMINANCE,” contains raw data from the Q-FISH analysis. The first four columns are “Chromosome,” “1o2o,” “Metaphase,” and “Tails,” respectively. The column “Chromosome” is associated with the numbers of the examined chromosomes (Chr01–Chr23), column “1o2o” describes which chromosome pair was analyzed (values a or b), column “Metaphase” denotes the number of metaphases (values 1–10), and column “Tails” refers to the four ends of the chromosomes (values p1, p2, q1, q2). If data were missing or values are close to background fluorescence, the code ND (Not detected) was used. Data from Q-FISH fluorescence intensities were imported manually for this version, but our goal is to automate the process in the future versions.

The fifth sheet, “TL,” is used to calculate TL (in bases)as derived from Image J and imported to “ILLUMINANCE” sheet. TL calculations use fluorescence intensities data from the sheet “ILLUMINANCE” and the cell series included in the sheet “PARAMS.” ND values from the “ILLUMINANCE” sheet are represented as blanks cells.

**FIGURE 2:**
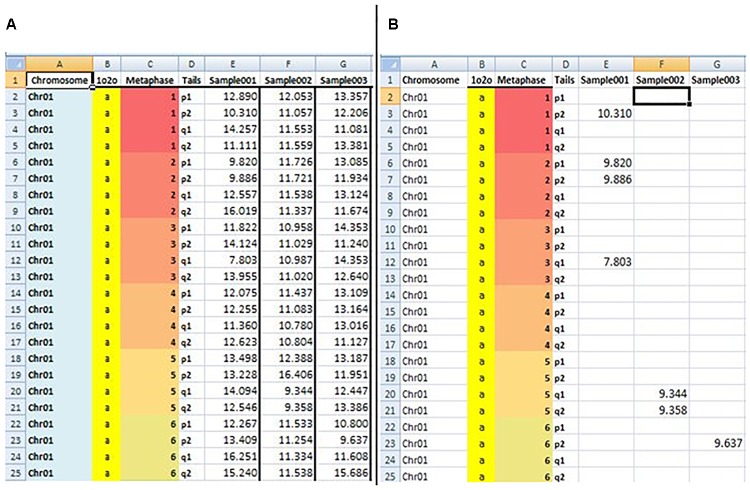
Snapshot of BIOTEL worksheets with estimated raw data of the TL **(A)** and TLS (TL < 20th percentile) **(B)**. In the **(A,B)** the first four column address the TL and TLS values by indexing from metaphase, chromsome, pair, and tail. From fifth column onwards values of TL **(A)** and TLS **(B)** of an individual were presented.

The sixth sheet, “TLS,” is used to measure short telomeres. In this sheet, auser-defined EXCEL formula was developed to detect short telomeres (TL > minimum and TL < 20th percentile) from the “TL” sheet data. The formula includes the functions IF, GESTEP (), CHOOSE (), and PERCENTILE (). An example of estimated data from the fifth and sixth sheet “TL” and “TLS” is shown in Figure 2.

The seventh sheet, “SYNOPSIS,” summarizes descriptive statistics of TL and TLS data as percentiles in 5% intervals ranging from 0 to 100. Data from TL or TLS measurements of all participants were analyzed. The eighth sheet, “PIVOTS,” summarizes results from the “SYNOPSIS” sheet in a pivot form vs. age group, sex or other variables of interest (e.g., use of drugs or vitamin supplements).

The ninth sheet, “CALCULUS,” is used to produce TL and TLS statistics, graphs and diagrams based on an individual’s data. In “CALCULUS,” the user can obtain a variety of information such as a text description of statistics, detailed description per pair of chromosomes, and grouped frequencies of telomeres. In this sheet, percentile data of TL and TLS values vs. age groups, which were used for biological age estimations and the fitting information (coefficients, R^2^ goodness of fit), is presented. A list of produced BIOTEL diagrams in “CALCULUS” sheet can be found in Supplementary Material (Supplementary Figures S1–S5).

The tenth sheet, “COMPARATIVE,” presents repetitive calculations of an individual’s TL measurements. In the same sheet, the rates of TL or TLS changes between two or more measurements are also presented.

#### Coding of Results

Identification and reference codes were used to handle patient data. Identification codes were produced for confidentiality and recoding purposes. Reference codes were based on the consecutive series of the participants included in the database.

As an example of patient coding, assume that the 12th sample with the first name “SMITH,” surname “JOHN,” date of birth (12/1/1970), and sex “male” with fir 1st telomere examination was coded. For this sample, the reference Code is “Sample012” and the identification code based on personal and examination data could be “SMIJOH1970MR1.”

Another coding system was applied for the personal data and estimated telomere, and short telomere data. Code “Sample XXX,” for example, was related to the range of EXCEL cells that included TL measurements of patient XXX (where XXX is the number of the consecutive series). Code Sample XXXS was assigned to the TLS data of patient XXX.“Sample XXX” and “Sample XXXS” were produced using the “Name Manager” feature of EXCEL.

#### Non-automated Procedures (User Interventions)

Because BIOTEL is a semi-automated spreadsheet, the user must perform some specific tasks. The first task is the definition of the EXCEL data ranges into useful and easily handled reference names, to facilitate the analysis.

Second task, following the first task, is the use of Replace (all) the previous reference names with a new one, for providing the TL (TLS) statistics of a new entry.

## Results

### Descriptive Telomere Statistics

**FIGURE 3:**
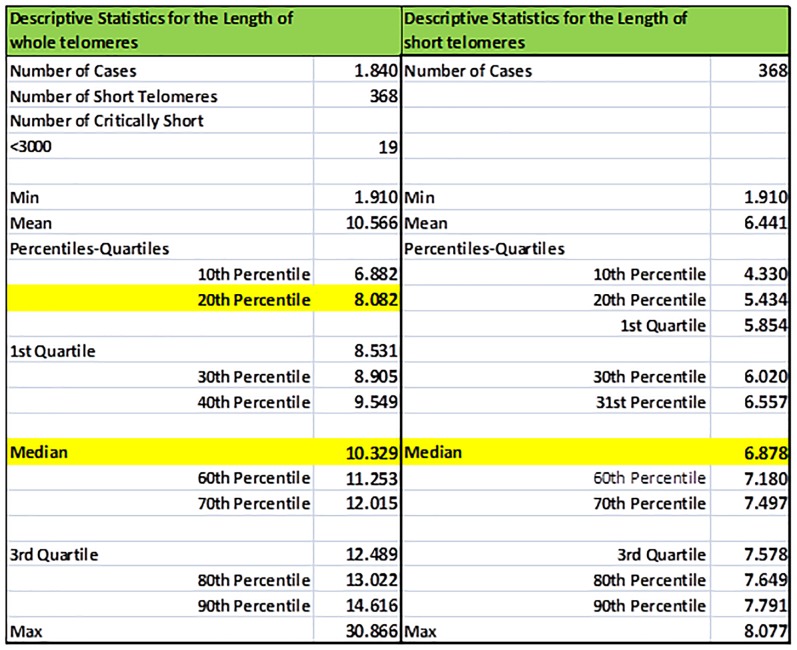
Sample basic descriptive statistics (percentiles, quartiles, min/max, mean) of the whole (TL) and short (TLS) telomeres (<20th percentile) of a selected individual. In our example statistics of the individual re-coded as Sample005 is shown.

Statistics of TL and TLS of an individual are illustrated in Figure 3, including percentiles and quartiles of the whole telomere genome and the short telomeres (<20th percentile). Values were estimated using the EXCEL functions: COUNT (Sample XXX), COUNTIF (Sample XXX; limit), AVERAGE (Sample XXX), MEDIAN (Sample XXX), QUARTILE (Sample XXX; a) where a equals to 1 or 3, MIN (Sample XXX), MAX (Sample XXX), and PERCENTILE (Sample XXX; b) where b ranges from 0.0 to 1.0. Corresponding descriptive statistics were produced for short telomeres by replacing Sample XXX datasets with Sample XXXS.

**FIGURE 4:**
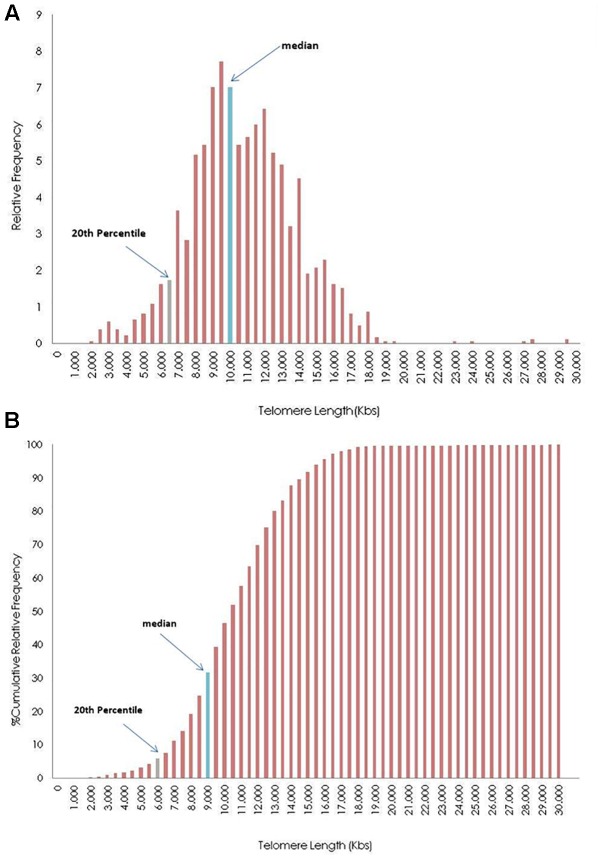
Distribution of telomere length (TL) in 0.5 kilobases (Kb) intervals of the whole genome frequencies **(A)** and cumulative frequencies **(B)** of a participant. The blue bar represents the group where the median values of the TL is in frequencies bar, and gray bar shows the group where the median of 20th percentile of telomeres (upper limit of short telomeres) is located.

Figure 4 shows the distribution of telomere length of an individual, after grouping TL in 0.5 kb intervals. TL was estimated from 1840 measurements (23 chromosomes ×2 pairs of chromosomes ×4 tails ×10 metaphases). The upper histogram demonstrates the relative frequency of TL, while the lower histogram demonstrates the relative cumulative distribution. Histograms were produced using the function FREQUENCY (Sample XXX; c), where c is the upper limit of each telomere group (e.g., 1500, 2000, 2500). Marked bars denote the TL median and 20th percentile.

### Biological Age Estimation

**FIGURE 5:**
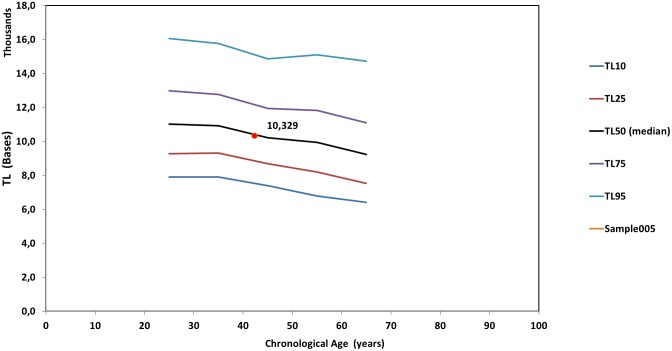
Reference lines [telomere length (TL) vs. chronological age] based on the whole genome measures. The red dot denotes the median TL of an individual. TL10 through TL95 correspond to the respective percentiles of TL of the population statistics.

Figure 5 is a line diagram of telomere length values of a reference population vs. chronological age, where an individual’s median TL is presented with a red dot. The chronological age was estimated from the date of sampling minus the date of birth. Corresponding curves based on the distribution of TLS were also produced (not shown). Point estimation of a person’s biological age was calculated through a linear fitting on the nearest reference line according to if the person is over or under their age group median line. Importantly, sex did not significantly (*p* > 0.05) affect TL and TLS percentile values.

**FIGURE 6:**
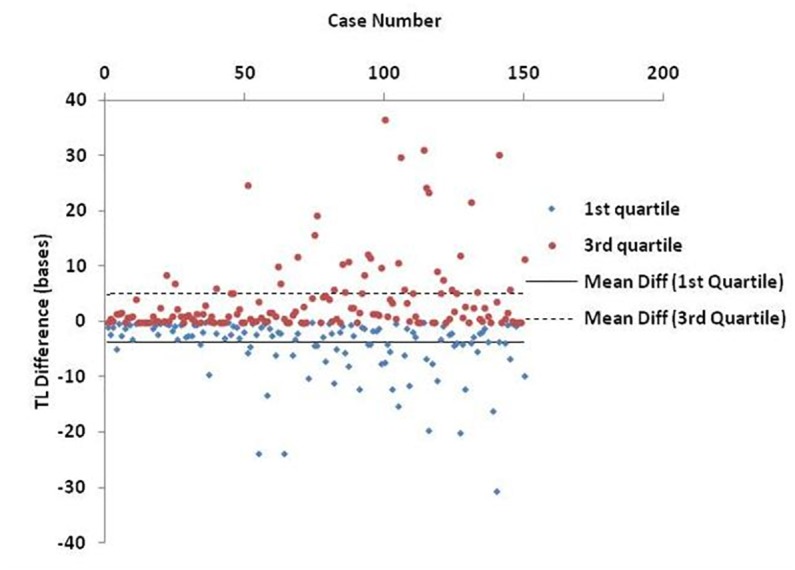
Scatter plot of estimated differences in telomere length (TL) quartiles. Each point denotes the individual’s difference in TL as calculated from BIOTEL and IBM SPSS Statistics 23.0. Lines refer to mean difference (mean diff) of 1st quartile (solid line) and 3rd quartile (dashed line).

Finally, Figure 6 shows an estimation of the BIOTEL accuracy. TL quartiles of 150 participants from the TLDP database were calculated from raw TL data using BIOTEL and IBM SPSS Statistics 23.0. The median TL calculated by BIOTEL and SPSS based on Tukey’s Hinges method were identical (average = 0, *SD* = 0). First quartile measures in BIOTEL were underestimated (mean of differences = -3.9, median = -1.8), while the opposite was shown in third quartile measurements (mean differences = 4.9, median = 1.4).

## Discussion

In this study, we present BIOTEL version 2.4, a spreadsheet developed to calculate TL statistics. BIOTEL is a standalone program based on the utilities provided by MS EXCEL. The creators of BIOTEL had not applied any VBA routine to avoid compilation issues in future upgrades. BIOTEL was developed in MS EXCEL 2010, but it has been tested and was found to function in MS EXCEL 2013 correctly. The software can be characterized as user-friendly, due to the EXCEL platform used and it can quickly produce an individual’s or group’s TL statistics. Names of the datasets were created in a form that can be replaced using the “Replace” feature of MS EXCEL, providing statistics of another person.

BIOTEL has further applications including, bar charts of the extremely short (<3 kb) and extremely long (>20 kb) telomeres, bar charts demonstrating the position of personal measures relative to the population median TL or TLS, a graph of biological vs. chronological age, and median TL per chromosome and telomere end. Furthermore, new graphs, statistics, and mathematical approaches could be included in the program, as described in future updates. Additionally, the user could easily add new features (graphs, reports, statistics) or modify the existent ones (scales, limits, colors, and other).

BIOTEL was created to improve the analysis of telomere data, which can be used to estimate biological age. Currently, there is a debate on the estimation of biological age using TL. Indeed, extensive manuscripts and reviews have studied the association between TL and outcomes in various pathologies such as cardiovascular diseases (Panayiotou et al., 2010; Jylhävä et al., 2017). However, there is no definitive way to estimate the biological age. In this study, we estimated biological age using nomograms produced from the BIOTEL TL analysis. In this approach, the position of the median short TL is placed in a nomogram of age vs. TLS. Biological age estimates can be obtained by calculating the inverse linear relationship of short TL vs. age, taking into consideration two hypothesis. The first hypothesis is that the values under the median telomere nomograph indicate that the biological age of the individual is higher than their chronological age. The second hypothesis is that calculation of age vs. TLS equation is made using the nearest percentiles curves, above or below TL.

The accuracy of BIOTEL results was assessed by comparing them with those obtained by the widely used statistical package, IBM SPPS Statistics. As presented in Figure 6, the calculations obtained by the two programs did not differ in median estimations. However, the differences in 1st and 3rd quartile between spreadsheet and statistical program showed a systematic deviation of overestimation in 1st quartile and underestimation in the 3rd quartile. Those differences cannot be considered as critical because they do not exceed the 40 bases per measurement and are probably related to the program(s) algorithms or numerical precision of estimated TL/TLS like significant digits and numbers rounding.

In this study, we implemented BIOTEL using data from the TLDP, an under-development telomere database. The TLDP is useful for research purposes and is fully compatible with other specialized statistics platforms, such as IBM SPSS Statistics. As of June 2018, TL statistics were calculated for 150 participants, and with time more individuals will be included. Additionally, three datasets of research interest have been included: (a) a group with pesticide exposure (occupational exposure), (b) a group with systematic use of vitamin supplements, and (c) a group with long-term use of narcotics, mainly cannabis. Preliminary analysis of these three groups were performed using BIOTEL, and their results have been reported (Alegkakis et al., 2017; Vakonaki et al., 2017, 2016). Interestingly, in 4 out of five individuals with regular usage of cannabis and opiates, had small TLS values that were in the first 10th percentile (Vakonaki et al., 2017).

Currently, BIOTEL is a freeware spreadsheet. In the next versions, specific additional tasks need to be considered. To improve the user interface, we are preparing a user’s manual and a fully automated report of an individual’s TL data. For TL analysis, we are developing a semi-automated method to update the TL curves with recently analyzed data. Specifically, an application with more mathematical curves on the TL vs. age fitting (TL vs. nomograms), and the use of other approaches based on the TL distribution is under development. Ultimately, the goal of BIOTEL is to handle TL data automatically and facilitate the whole process from Q-FISH analysis to statistics report, including pattern recognition, image processing, and data analysis. Q-FISH allows the assessment of critically and extremely short telomeres (<3 kb), fused chromosomes, detected as critically long telomeres, and provides an overview of the telomeric profile. Therefore, it remains the most reliable method to generate accurate diagnostic results, with little margin of error.

Importantly, BIOTEL is the first attempt to manipulate and analyze raw TL data and use it for the estimation of biological age. Although many studies have presented TL data for different populations (Sanders and Newman, 2013; Lapham et al., 2015) and there is a specialized company (Life Length, Madrid, Spain) focusing on this issue, we did not find any references to TL databases or statistics. Considering that TL data can be utilized to estimate biological age, methods that facilitate the analysis of these data can significantly improve our understanding of aging and factors that affect it.

## Conclusion

BIOTEL is an ongoing computer program (worksheet) used for generating TL statistics from raw data, which can be used for research purposes. This worksheet was implemented with data from the TLDP and was designed to provide both individual and grouped data. BIOTEL is user-friendly, provides communication with statistics platforms, is upgradable, and has several applications. It can be used for both research and commercial purposes -under license restrictions-, including research analyzing TL and estimating biological age.

## Availability of Data and Material

The datasets generated and/or analyzed during the current study are not public available due to ethical reasons but are available from the corresponding author on reasonable request.

## Ethics Statement

This study was carried out under the recommendations of Ethics Committee of the University Hospital of Crete. The sampling procedures were defined according to the ethical guidelines of the Ethics Committee of the University Hospital of Crete (approval no. 1019/18/07_12_2016) confirming informed consent was obtained from all participants for this study. All work has been conducted under the Declaration of Helsinki (1964).

## Author Contributions

DT supervised participant selection, and the software development was based on his idea. GT helped during the obtaining of participants’ medical histories and clinical evaluations, contributed to participant selection and manuscript preparation. AA created and developed the software. PF, EV, and MT performed the Q-FISH measurements, providing fluorescence data, and were responsible for the biological information. ES and DN contributed to study design, data interpretation as well as the preparation and editing of the manuscript. AT, participated in study design, data interpretation, and manuscript preparation as well as supervised the study.

## Conflict of Interest Statement

The authors declare that the research was conducted in the absence of any commercial or financial relationships that could be construed as a potential conflict of interest.

## Footnotes

1 https://imagej.nih.gov/ij/

## Abbreviations

PNA: Peptide nucleic acid
Q-FISH: Quantitative Fluorescence *in situ* Hybridization
TL: Telomere Length
TLS: Telomere Length of short (<20th Percentile)telomeres
TLDP: Telomere Length Database Project
VBA: Visual Basic for applications
