# Supplementary material for: Developing BIOTEL: A Semi-Automated Spreadsheet for Estimating Telomere Length and Biological Age
Source: Front Genet. 2019 Feb 19;10:84. doi: 10.3389/fgene.2019.00084 (PMC6389611; doi:10.3389/fgene.2019.00084)
Supplement: Supplementary file 1 [file Table_1.DOCX]

**Legends for Supplementary Figures**

**Figure 1S.** Frequencies of Telomere Length (TL) per 0.5 Kbases

**Figure 2S.** Barchart of an individuals TL or TLS statistics and population’s TL or TLS medians

**Figure 3S.** Barchart of extremely low (<3% percentile) and extremely high (>97^th^ percentile) of an individual’s TL data

**Figure 4S.** Scatterplot of estimated biological age with chronological (actual) age

**Figure 5S.** Snapshot of BIOTEL’s usage tips.

**Table 1S.** Linear fitting of TLS with actual age for 10^th^, 30^th^ and 60^th^ percentie

| **Percentile** | **Beta** | **R2** |
| --- | --- | --- |
| 10th | -61.15 | 0.348 |
| 30th | -60.37 | 0.398 |
| 60th | -62.21 | 0.388 |
