# Supplementary figures and images for: Developing BIOTEL: A Semi-Automated Spreadsheet for Estimating Telomere Length and Biological Age
Source: Front Genet. 2019 Feb 19;10:84. doi: 10.3389/fgene.2019.00084 (PMC6389611; doi:10.3389/fgene.2019.00084)

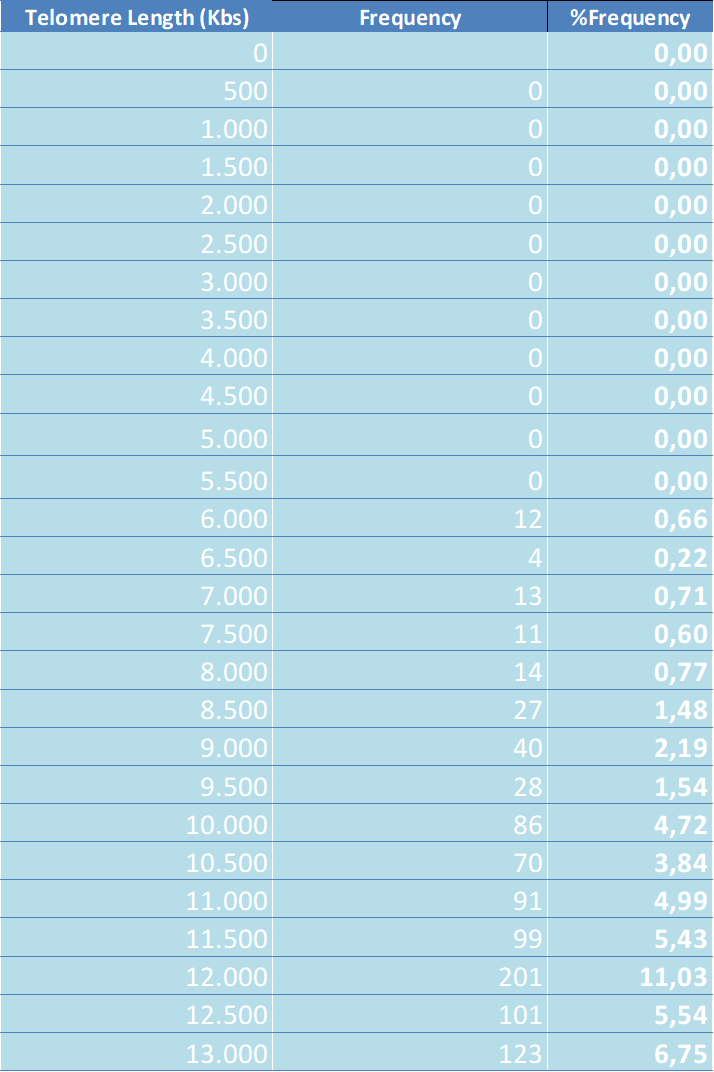

Supplement: Supplementary file 2 [file Image_1.TIF]

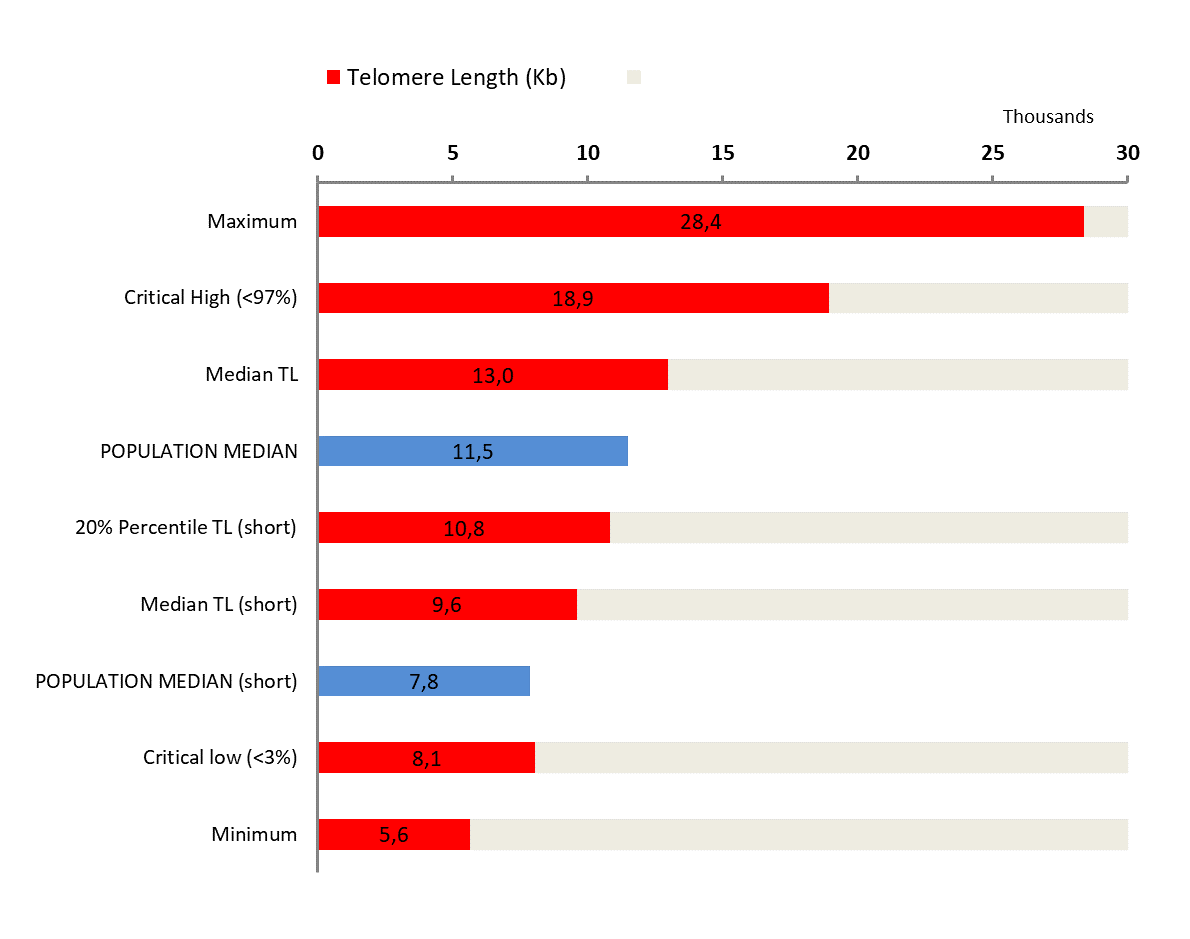

Supplement: Supplementary file 3 [file Image_2.TIF]

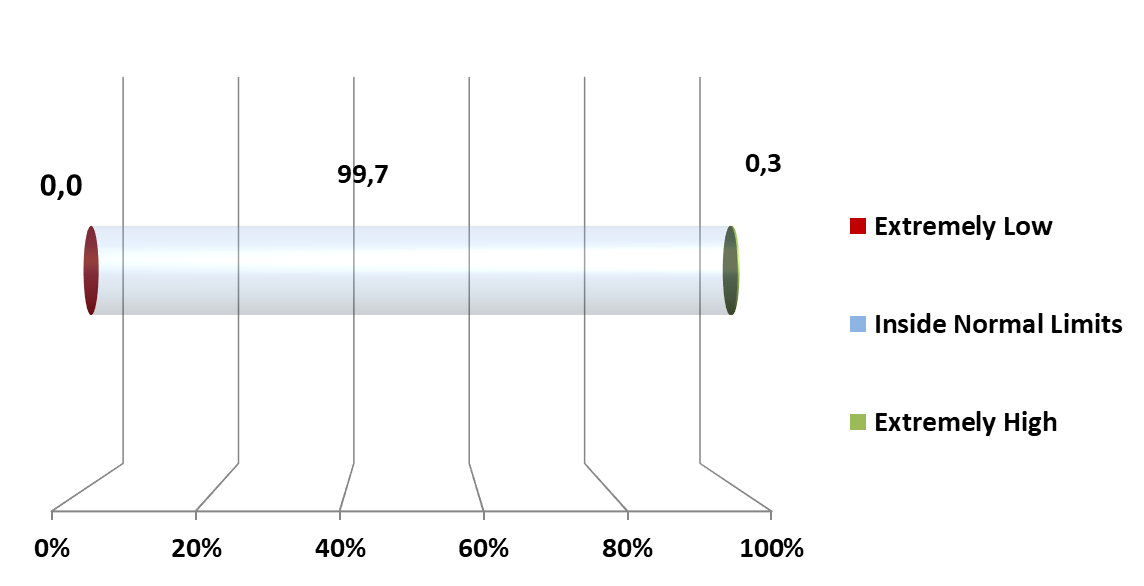

Supplement: Supplementary file 4 [file Image_3.TIF]

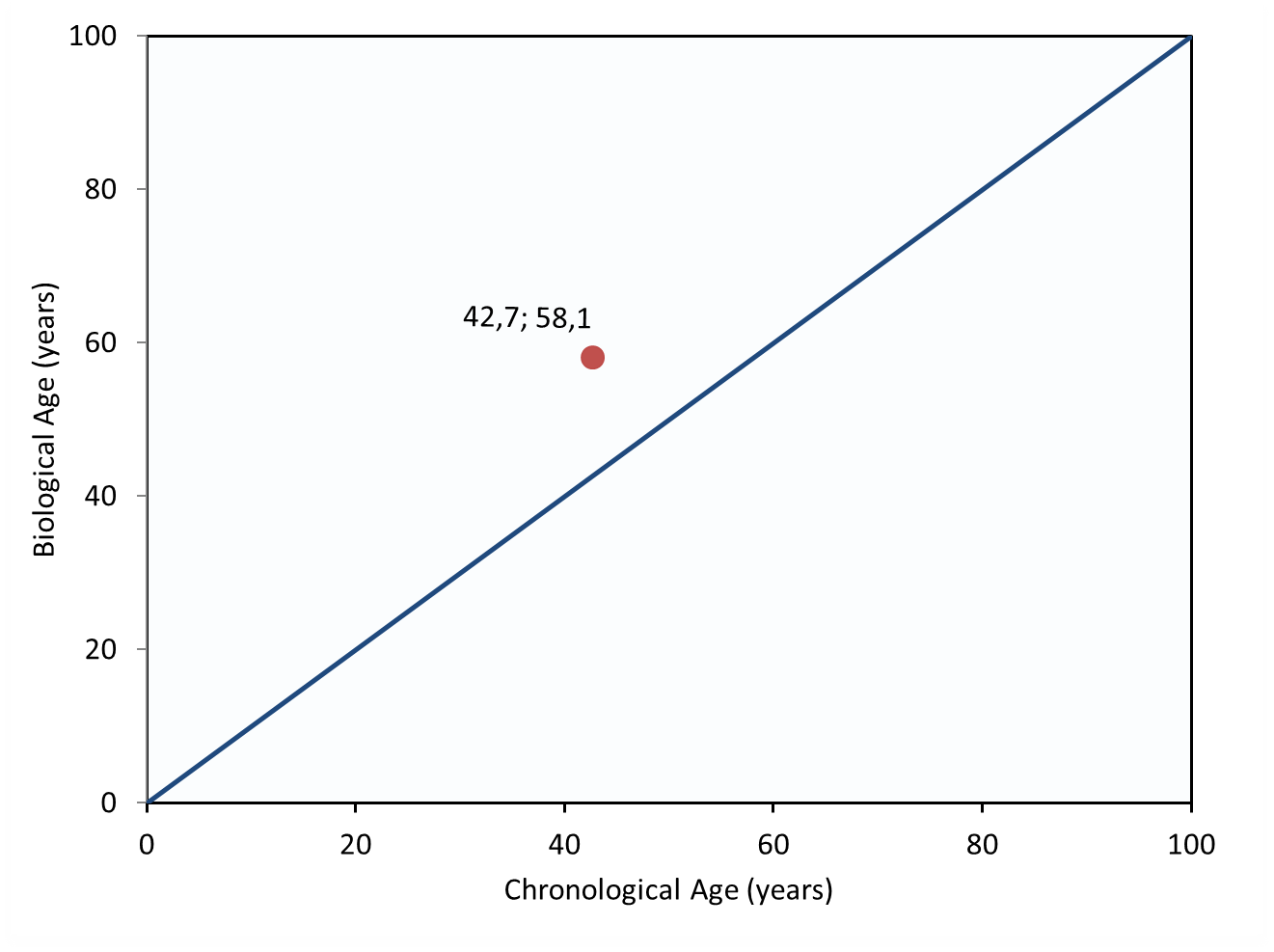

Supplement: Supplementary file 5 [file Image_4.TIF]

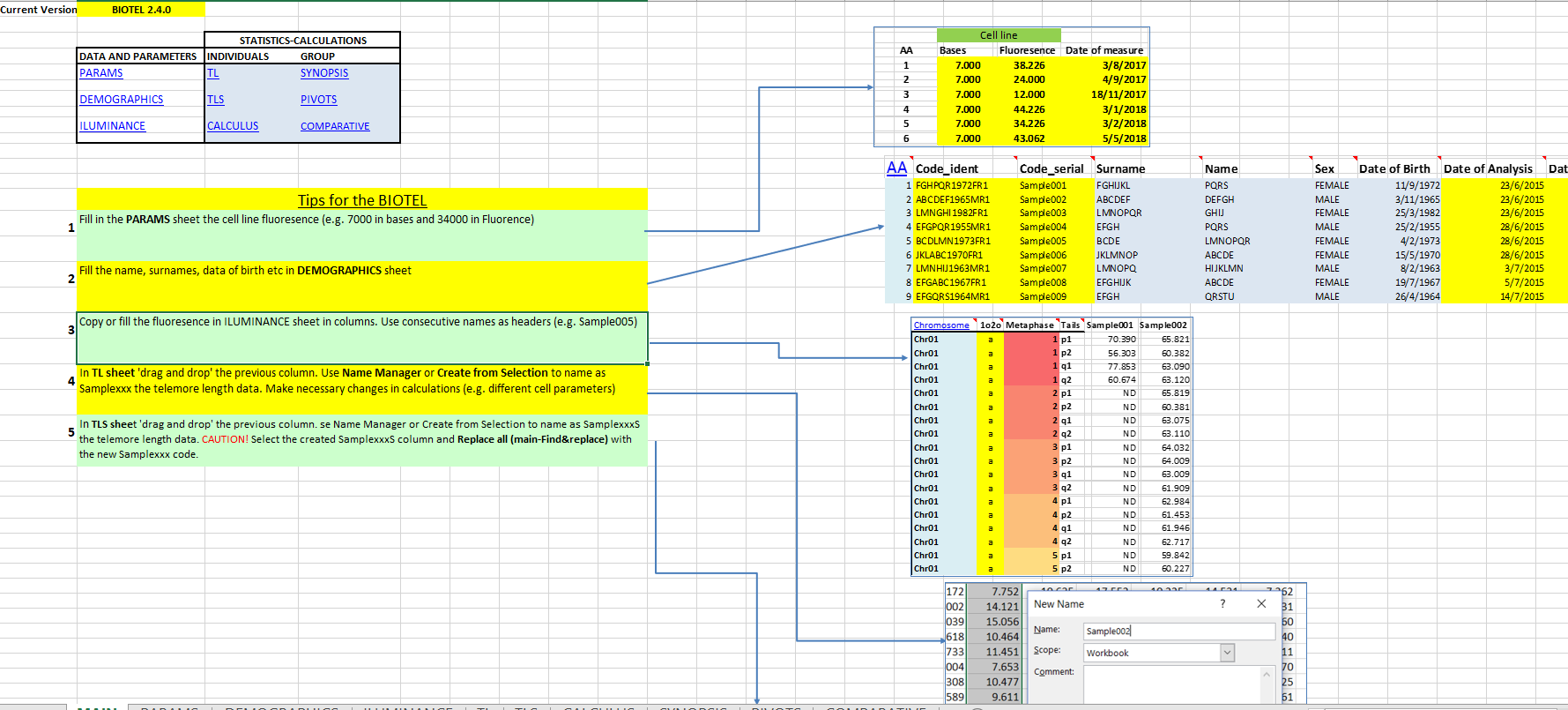

Supplement: Supplementary file 6 [file Image_5.TIF]
